# Large-scale production of functional human lysozyme from marker-free transgenic cloned cows

**DOI:** 10.1038/srep22947

**Published:** 2016-03-10

**Authors:** Dan Lu, Shen Liu, Fangrong Ding, Haiping Wang, Jing Li, Ling Li, Yunping Dai, Ning Li

**Affiliations:** 1 The State Key Laboratory for Agro-biotechnology, China Agricultural University, Beijing, China; 2 Shanghai Institute of Medical Genetics, Shanghai Children’s Hospital, Shanghai, China

## Abstract

Human lysozyme is an important natural non-specific immune protein that is highly expressed in breast milk and participates in the immune response of infants against bacterial and viral infections. Considering the medicinal value and market demand for human lysozyme, an animal model for large-scale production of recombinant human lysozyme (rhLZ) is needed. In this study, we generated transgenic cloned cows with the marker-free vector pBAC-hLF-hLZ, which was shown to efficiently express rhLZ in cow milk. Seven transgenic cloned cows, identified by polymerase chain reaction, Southern blot, and western blot analyses, produced rhLZ in milk at concentrations of up to 3149.19 ± 24.80 mg/L. The purified rhLZ had a similar molecular weight and enzymatic activity as wild-type human lysozyme possessed the same C-terminal and N-terminal amino acid sequences. The preliminary results from the milk yield and milk compositions from a naturally lactating transgenic cloned cow 0906 were also tested. These results provide a solid foundation for the large-scale production of rhLZ in the future.

There is no doubt that breast milk provides an ideal source of nutrition for infants, as well as promoting rapid growth and conveys more advantages than formula, as proteins in breast milk not only provide a well-balanced source of amino acids for nutritional needs, but also simultaneously aids in the defense against infections, enhances immune function, and promotes development of gut function^1^,^2^. To improve the nutritional content of infant formulas, attempts have been made to add human proteins, such as *α*-lactalbumin^3^, lactoferrin^4^,^5^, and lysozyme^6^, to bovine milk by producing genetically modifying dairy cows and to remove the major allergenic bovine protein β-lactoglobulin^7^,^8^.

Lysozyme is known to exert a wide range of antimicrobial activities against pathogenic bacteria and viruses, and has been widely used in food, pharmaceuticals, and clinical treatments, such as oral health care and antibiotic therapy^9^,^10^. However, most lysozyme for pharmaceutical use is purified from egg whites and thus may induce egg allergy among those sensitive to lysozyme^11^. Considering pharmaceutical security and market demand, large amounts of recombinant human lysozyme (rhLZ) should be produced. An alternative means of producing rhLZ is through a mammary gland bioreactor system in transgenic farm animals expressing rhLZ in the mammary gland. Systemic and thorough work on transgenic goats provided pioneering achievements for the development of rhLZ transgenic livestock. A large amount of rhLZ can be obtained and purified from transgenic goat milk; the effects of rhLZ goat milk both *in vitro* and *in vivo* have also been tested. Feeding pigs with rhLZ transgenic milk produced by goats can improve gut microbiota and morphology, increase the expression of the anti-inflammatory cytokine TGFβ1, and change the composition of serum metabolites, all of which were reported to improve the health status of pigs^12^,^13^,^14^,^15^. Additionally, milk containing rhLZ can provide a rapid recovery from diarrhea in pigs infected by pathogenic bacteria^16^. The physical, chemical, and physiological characteristics of rhLZ have been extensively reported. rhLZ expressed in milk from both transgenic goats and cows has a similar pH and temperature stability to those of natural lysozyme *in vitro* and the presence of rhLZ does not affect the composition of milk^6^,^17^,^18^,^19^. Moreover, lysozyme may prevent the occurrence of mastitis by killing pathogenic bacteria, such as *Staphylococcus aureus*, which can benefit the health of cows as well as reduce losses to the dairy industry^20^,^21^.

Here, we focused on generating rhLZ transgenic cows due to their large milk yield being more ideal as bioreactors for the production of large quantities of recombinant proteins. Although transgenic cows have been generated with different rhLZ constructs^6^,^20^, the low expression of rhLZ in the mammary gland of those cows has limited its commercial application. The results of our previous studies showed that the vector pBAC-hLF-hLZ-Neo can efficiently express rhLZ both in mice^22^ and pigs^23^. In consideration of bio-safety issues, we modified the vector pBAC-hLF-hLZ-Neo and removed the marker gene neomycin *in vitro*. Using this marker-free vector, we generated seven transgenic cloned cows that produced rhLZ in milk at concentrations of up to 3149.19 ± 24.80 mg/L. These transgenic cows produced high concentrations of rhLZ in milk, thereby laying a solid foundation to produce rhLZ via the mammary gland bioreactor system and also to improve milk quality and resistance to mastitis.

## Materials and Methods

### Construction of the marker-free plasmid pBAC-hLF-hLZ

The plasmid 706-Cre (Gene Bridges GmbH, Heidelberg, Germany), which can express Cre recombinase, was transformed into competent DH10β cells that contain the pBAC-hLF-hLZ-Neo plasmid carrying a floxed (flanked by loxP sites) selection marker. After transformation, 1 mL of lysogeny broth (LB) medium was added to each tube, which was then incubated at 30 °C for 1.5 h while shaking at 220 rpm. The cells were streaked onto LB plates containing 50 μg/mL of kanamycin and 5 μg/mL of tetracycline. The plates were incubated at 30 °C for 24 h. A single colony was picked and grown in 1 mL of LB medium at 30 °C for 3 h and then incubated at 37 °C overnight. After preparing the plasmid DNA, part of the DNA was re-transformed and the recombined plasmid was identified by polymerase chain reaction (PCR) analysis.

### PCR analysis

Successful transfer of the rhLZ gene to the transgenic cloned cows was confirmed by PCR analysis. The P-HLZ-637F/R primer pair (P-HLZ-637-F: 5′-TTATACACACGGCTTTAC-3′; P-HLZ-637-R: 5′-CAGCATCAGCGATGTTATCT-3′), which can amplify a 637 bp product, was used to confirm successful transfer of the rhLZ gene to the transgenic cloned cows and also to amplify the probe for Southern blot analysis. The P2F/R primer pair (P2F: 5′-TGCTTTGTTTGTATTGAGGGTC-3′; P2R: 5′-CCAGGAACAAACTTACGGAG-3′) was used to identify the recombined plasmid pBAC-hLF-hLZ. The size of the product of an unrecombined plasmid was 2541 bp, while that from a recombined plasmid was 800 bp.

### Cell culture and nuclear transfer

The NucleoBond bacterial artificial chromosome (BAC) 100 Kit (Macherey-Nagel GmbH & Co. KG, Düren, Germany) was used to purify the plasmid pBAC-hLF-hLZ after linearization with the restriction endonuclease NotI. Primary bovine fetal fibroblasts were isolated from a 46-day-old Holstein cow fetus. The linearized plasmid DNA fragment was transfected into embryonic fibroblasts cells using Amaxa Nucleofector reagent (Lonza Group AG Basel, Switzerland), following program T-016. Cells were dispersed by limiting the dilution to a cell concentration of 300 cells/dish (10 cm^2^) 48 h after transfection. The cells of each clone were subsequently expanded into a 48-well cell culture plate and cultured for another 48 h before being passaged into a 12-well cell culture plate. One-sixth of the cells from the 12-well cell culture plate was used for PCR analysis.

Somatic cell nuclear transfer (SCNT) was performed in accordance with previously reported methods^24^,^25^. The transgene-carrying cell colonies (donors) were transferred into enucleated oocytes for the production of reconstructed embryos using the ECM® 2001 Electro Cell Manipulation System (BTX, San Diego, CA, USA). Day 7 blastocysts were collected for future transplantation. The vitrification method was used for embryo cryopreservation. All blastocysts were transferred after freezing/thawing. A total of 241 blastocysts were transferred into 161 recipient Chinese Luxi yellow cows. For each recipient, we transferred 1–2 transgenic cloned blastocysts. Pregnancy was detected by ultrasonography at 60 days and 180 days post-transfer. All experiments were performed in accordance with relevant guidelines and regulations; the Institutional Animal Care and Use Committee of China Agricultural University approved this research.

### Southern blot analysis

The plasmid pBAC-hLF-hLZ and the DNA samples extracted from ear samples of transgenic and wild-type (WT) cows were digested overnight with the restriction enzyme EcoRI. The digoxigenin-labeled probe was amplified using the primer pair P-HLZ-637F/R. After agarose gel electrophoresis for 4 h, DNA was transferred to a nitrocellulose filter for blotting. The nitrocellulose membrane was hybridized with a probe for 18 h and incubated with antibody for 0.5 h. The size of positive bands was expected to be about 3.3 kb. The reagents used for Southern blot analysis were purchased from Roche Diagnostics GmbH (Mannheim, Germany).

### Quantitative PCR (Q-PCR)

Q-PCR was used to detect the transgene copy number. The primer pair P-hLZ-112F/R (P-hLZ-112F: 5′- TGCTGGGTGCCTGAGATTCA-3′; P-hLZ-112FR: 5′-GTTCAAAATGGGAAATAACTGG-3′), which can amplify a 112 bp product, was used to calculate the transgene copy number in the transgenic cows. The primer pair bovine myostatin F/R (bovine myostatin F: 5′-TCCGTCCTGGCGTGGTAG-3′; bovine myostatin R: 5′-GCTATCAGACAACTTTTGCCCAAG-3′), which can amplify a 122 bp product from the myostatin gene (GenBank: JQ711180.1), was used as an internal control. Each PCR amplification was performed in a 20 μL reaction volume containing 1 μL of template DNA (10 ng/μL), 0.3 μL of each primer, 10 μL of Power SYBR Green Mix, and 8.4 μL of distilled deionized water (ddH_2_O) performed under the following conditions: initial denaturation at 95 °C for 10 min, then 40 cycles of denaturation (95 °C for 10 s), annealing (60 °C for 10 s), and extension (72 °C for 10 s), followed by 95 °C for 5 s, 65 °C for 1 min, and then 97 °C continuously to generate a melting curve. A gradient dilution of the vector DNA mixed with WT DNA (10 ng) was used to produce a standard curve, with SYBR green functioning as the fluorescent dye. All PCR reactions were performed using the Roche LightCycler 480 System (LC 480; Roche Diagnostics, Basel, Switzerland).

### Collection of transgenic milk

To induce lactation, we intramuscularly injected medroxyprogesterone acetate (25 mg/kg/day) and estradiol benzoate (7.5 mg/kg/day) into four transgenic cloned cows (0814, 0827, 0906 and 0910) at the age of eight months for seven days. Milk samples were collected for more than two weeks from day one of lactation.

### Milk yield and milk compositions

Transgenic cloned cow 0906 was milked twice daily at 0730 and 1530 h, and milk yields were recorded. Milk samples were collected on day 1 to day 17 during each data collection period. The percentage (w/vol) of fat, protein, lactose, and dry matter were determined using a MilkoScan 4000 (Foss, Hillerod, Denmark).

### Sodium dodecyl sulfate polyacrylamide gel electrophoresis (SDS-PAGE) and western blot analysis

For SDS-PAGE, milk protein samples were separated on 12% Tris-glycine polyacrylamide gels under denaturing and reducing conditions, and the protein content was quantified by dying with Coomassie brilliant blue. For western blot analysis, the diluted milk samples were separated on 15% Tris-glycine polyacrylamide gels under denaturing and reducing conditions then transferred to polyvinyl difluoride membranes (Invitrogen Corporation, Carlsbad, CA, USA), which were incubated with a polyclonal anti-human HLZ antibody (dilution, 1:2,000; United States Biological, Inc., Swampscott, MA, USA) and a horseradish peroxidase-conjugated secondary anti-rabbit IgG antibody (dilution, 1:20,000; Sino-American Co., Beijing, China).

### Analysis of the rhLZ expression levels and activities

rhLZ expression levels were measured using a human lysozyme enzyme-linked immunosorbent assay (ELISA) kit (Biomedical Technologies, Inc., Stoughton, MA, USA). The concentrations of rhLZ in the milk of naturally lactating transgenic cloned cow 0906 over the first two weeks of lactation were measured using high performance liquid chromatography (HPLC) (see Supplementary Methods).

*Micrococcus lysodeikticus* cells (China General Microbiological Culture Collection Center, Beijing, China) were revived and prepared for gel diffusion and turbidimetric assays to assess the activity of purified rhLZ in the milk of transgenic cows. Briefly, 2.5 mL of *M. lysodeikticus* cell suspension at an absorbance at 450 nm (A_450_, 0.60–0.7) was loaded into a 4 mL cuvette as the substrate. Then, 100 μL of diluted milk samples or 100 μL of ddH_2_O was added to start the reaction. A_450_ was monitored at least for 5 min. One unit will produce a ΔA_450_ of 0.001 nm/min at pH 6.24 at 25 °C.

For the gel diffusion assay, the medium was prepared by mixing 100 μL of *M. lysodeikticus* suspension with 300 mL of solid culture medium, which contained 1.5% nutrient broth agar (Sigma–Aldrich Corporation, St. Louis, MO, USA). Samples were loaded onto 6 mm quantitative filter paper discs and the results were observed directly from the inhibition zones around the filter paper discs.

### Purification of rhLZ

Milk was centrifuged at 2500 rpm for 20 min at 4 °C to remove the fat. The skimmed milk was acidified to pH 4.6 to precipitate casein and centrifuged at 100,000 × *g* at 20 °C for 1 h. The purification procedure was performed using an ÄKTA pure system (GE Healthcare, Uppsala, Sweden). First, after equilibration in a column with equilibration buffer (20 mM phosphate buffer (PB), pH 8.2), samples were loaded into a HiScreen SP Sepharose FF column (GE Healthcare, Uppsala, Sweden; 4.7 mL) and the protein was eluted with a linear gradient of 0–1 M NaCl in 20 mM PB, pH 8.2. Then, an Ultracel-30 membrane (Millipore Corporation, Billerica, MA, USA) was used to concentrate the fractions containing rhLZ on the ÄKTA Crossflow automated cross flow filtration system (GE Healthcare, Uppsala, Sweden) After purification, the rhLZ was exchanged by 20 mM PB and the quantity and quality of the purified rhLZ was detected by SDS-PAGE.

### Molecular weight of purified rhLZ

The purified rHLZ was assayed by matrix-assisted laser desorption/ionization time of flight mass spectrometry (MALDI-TOF-MS) (Bruker Daltonics, Billerica, MA, USA) performed by Shanghai GeneCore Biotechnologies Co. Ltd. (Shanghai, China).

### N-terminal amino acid sequencing of purified rhLZ

The N-terminal amino acid sequence analysis was performed by Shanghai GeneCore Biotechnologies Co. Ltd. by the Edman degradation reaction using an automated Edman sequencer. The identified N-terminal amino acid sequence of purified rHLZ was compared with WT human lysozyme sequence data retrieved from the GenBank database (www.ncbi.nlm.nih.gov/genbank/) of the National Center for Biotechnology Information.

### C-terminal amino acid sequencing of purified rhLZ

The C-terminal amino acid sequence analysis was performed by Shanghai GeneCore Biotechnologies Co. Ltd. The samples were separately digested with trypsin, chymotrypsin, and endoproteinase Glu-C, and then analyzed by liquid chromatography tandem mass spectrometry (LC-MS/MS) using the Q Exactive™ Hybrid Quadrupole-Orbitrap Mass Spectrometer (Thermo-Fisher Scientific, Waltham, MA, USA). The identified C-terminal amino acid sequence of the purified rHLZ was compared with WT human lysozyme sequence data retrieved from the GenBank database.

## Results

### Construction of the marker-free plasmid pBAC-hLF-hLZ

**Figure 1:**
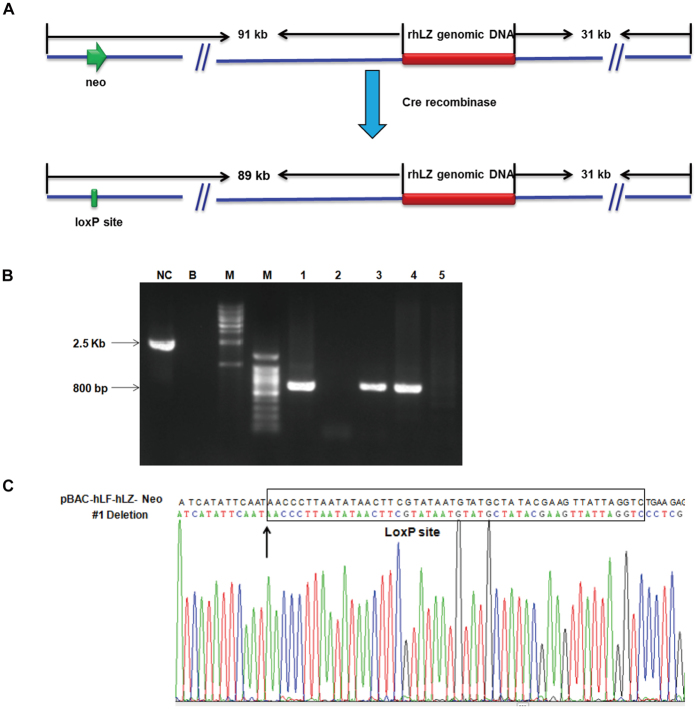
Construction of marker-free plasmid pBAC-hLF-hLZ. (**A**) A schematic of pBAC-hLF-hLZ. The pBAC-hLF-hLZ was constructed by modified the expression plasmid pBAC-hLF-hLZ-Neo. The kanamycin resistant cassette was removed by Cre recombinase and left a loxP sequense. (**B**) Identified the plasmid pBAC-hLF-hLZ by PCR. M, 1 kb or 100 bp DNA ladder; NC, plasmid pBAC-hLF-hLZ-Neo; B, water; samples 1–5 represent plasmids extracted from 5 bacteria colonies. (**C**) Sequencing result of plasmid #1. Sequence confirmation of modified BAC showing deletion occurred by compared to the plasmid pBAC-hLF-hLZ-Neo and a loxp sequence was retained after deletion.

The loxP-flanked marker gene Neo was removed *in vitro* from the plasmid 706-Cre, which can express Cre recombinase (Fig. 1A). After modifying the vector pBAC-hLF-hLZ-Neo, the final marker-free plasmid pBAC-hLF-hLZ was 125 kb in length. It contained a 4.8 kb human lysozyme genomic DNA fragment, an 89 kb 5′UTR and a 31 kb 3′UTR from hLF BAC. The recombined plasmids were confirmed by PCR analysis and the size of PCR product was expected be 800 bp (Fig. 1B). Of the plasmids from five different bacteria cell clones, three were positive and further confirmed by sequencing (Fig. 1C). As expected, only one loxP site remained and the Neo fragment was completely removed after recombination.

### Screening of positive transgenic cell colonies and generation of transgenic cloned cows by nuclear transfer

The expression plasmid pBAC-hLF-hLZ was transfected into bovine fetal fibroblast cell colonies 0901FFB and 1003FFB. After screening by single cell amplification, we isolated 298 0901FFB colonies and 334 1003FFB colonies, respectively. PCR analysis confirmed the presence of 17 positive cell colonies of which three were used as donor cells for nuclear transfer (#21, #27, and #14) (Table 1). As shown in Table 2, we produced a total of 2911 re-constructed embryos from three different colonies. Of these embryos, 32.4%, 41.7%, and 36.2%, respectively, successfully entered the blastocyst stage. In total, we transferred 241 transgenic cloned blastocysts into 161 recipients. When we assessed pregnancy by ultrasonography at 60 days, 25 recipients were found to be pregnant. However, ultrasonography at 180 days detected, that only 15 recipients recipients remained pregnant. Ultimately, we got ten alived new borned cattles and five stillbirths. Two of the ten alived cattles were died of lung infection after one month. All the surrogate mothers were healthy during the pregnancy and postpartum period.

### Transgene integration in the genome of transgenic calves

**Figure 2:**
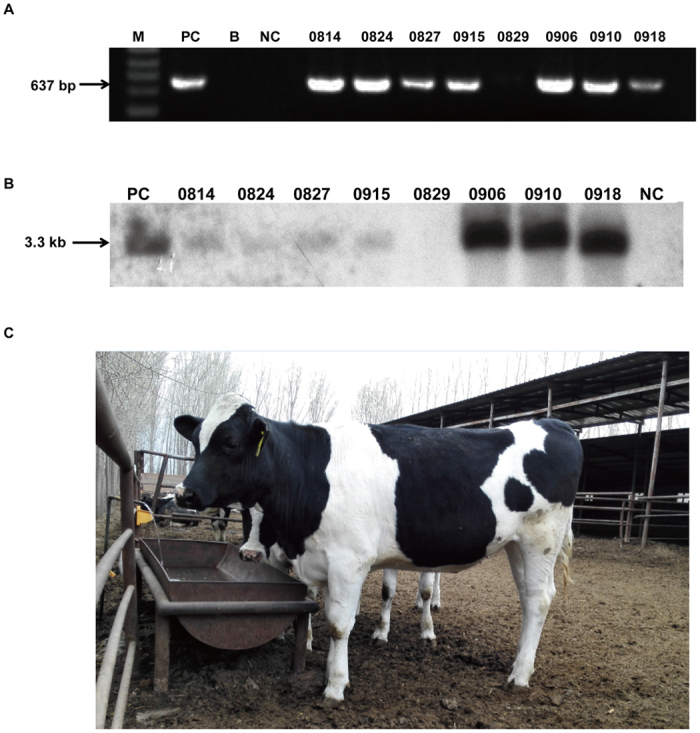
Integration of transgene in genome of transgenic cloned cows. (**A**) PCR analysis of transgenic cloned cows. M, 100 bp DNA ladder; PC, positive control vector pBAC-hLF-hLZ; B, water; NC, genomic DNA from WT cows; lanes 5–12, genomic DNA from transgenic cloned cows. (**B**) Southern blot identification of transgenic cloned cows. The bands were hybridized to a 637 bp specific probe that is complementary to a fragment of the hLZ gene. PC, plasmid vectors as the positive control; NC, genomic DNA from a WT cows; lanes 2–8, genomic DNA from transgenic cloned cows 0814, 0824, 0827, 0829, 0915, 0829, 0906, 0910 and 0918. (**C**) The image of transgenic cloned cows. The image shows the transgenic cloned cow 0906 at the age of 18 months.

The integration of the human lysozyme gene into the genome of the transgenic calves was confirmed by PCR and Southern blot analysis. The primer pair P-HLZ-637-F/R was used for PCR amplification and as a probe for Southern blot analysis. As shown in Fig. 2A, seven of the eight cloned cows were positive for expression of the transgene, while one cloned cow (#0829) was non-transgenic. This result was also further verified by Southern blot analysis (Fig. 2B). The band signals of different samples revealed a great deal of variation in the copy numbers of the transgenic cloned cows. As identified by Q-PCR, transgenic cows 0814, 0824, 0915, and 0927 cloned from cell clone #27 contained two copies of hLZ, and 0906 and 0910 contained eight and ten copies of hLZ, respectively. The image of transgenic cloned cow 0906 was shown in Fig. 2C. This picture was taken by yunping Dai on February 2015 at China Agricultural University farm.

### Expression of rhLZ in the milk of transgenic cloned cows

**Figure 3:**
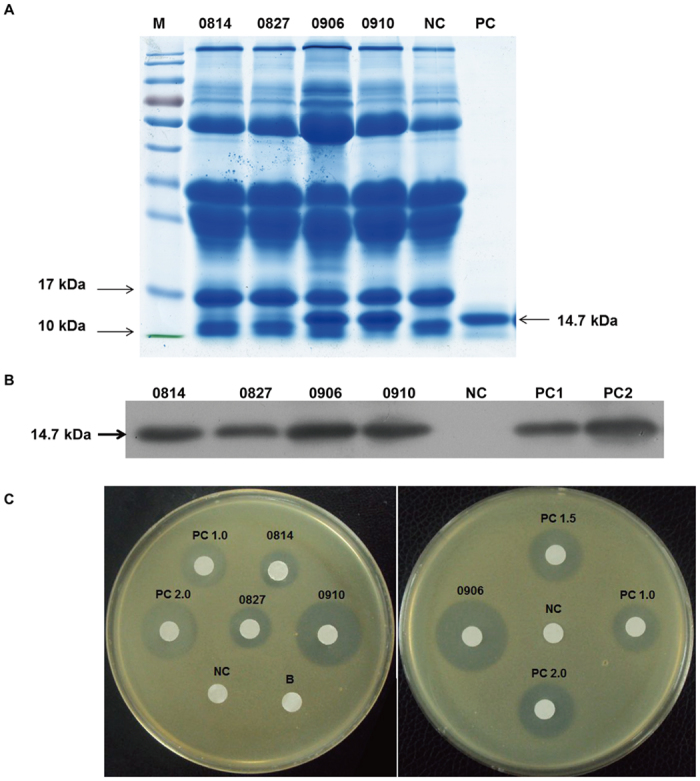
Expression of rhLZ in the milk. (**A**) SDS-PAGE of milk from transgenic cloned cows. Milk samples from transgenic-cloned cows were collected and detected by SDS-PAGE. PC, 1 μg commercial rhLZ as positive control; NC, milk from wild type cows. (**B**) Identification of rhLZ in the milk by western blot. PC1, 100 ng commercial natural hLZ standard; PC2, 300 ng commercial natural hLZ standard; NC, milk from WT cows; 0814 and 0827, 1 μL milk from transgenic-cloned cows; 0906 and 0910, diluted milk from transgenic-cloned cows (dilution, 1:10, 1 μL). C) Lytic activity of rhLZ against Mirococcus lysodeikticus. Dilution milk samples (dilution, 1:10, 10 μL) were added to 6 mm small white quantitative filters. PC1.0, PC1.5 and PC2.0 represent 1 μg, 1.5 μg and 2.0 μg commercial natural hLZ standard, respectively; NC, milk from WT cow.

Milk samples from four transgenic cows cloned from different cell colonies (0814, 0827, 0906, and 0910) and non-transgenic cows as negative controls were collected and analyzed by SDS-PAGE and western blot analysis. The size of human lysozyme is 14.7 kDa. SDS-PAGE of 0906 and 0910 showed obvious rhLZ bands, but the bands from 0814 and 0827 were not as clear (Fig. 3A). Western blot analysis showed positive bands for all of the samples from the transgenic cloned cows (Fig. 3B).

An ELISA was used to quantify the rhLZ expression levels in the milk of the transgenic cloned cows. As shown in Table 3, the rhLZ concentration in milk from 0906 was highest at 3149.19 ± 24.80 mg/L, while concentrations from 0814 and 0817 were much lower than those from 0906 and 0910, but were still around 400 mg/L, which is equal to the concentration of lysozyme in human milk. The enzymatic activities of rhLZ in the milk were also assessed using a gel diffusion assay to allow observation of the bacteriolytic activity of rhLZ milk *in vitro* (Fig. 3C). Compared with the positive controls, the content of rhLZ in milk from 0814 and 0817 was <1 μg, but that in milk from 0906 and 0910 was >2 μg, consistent with the ELISA results.

### rhLZ purification

**Figure 4:**
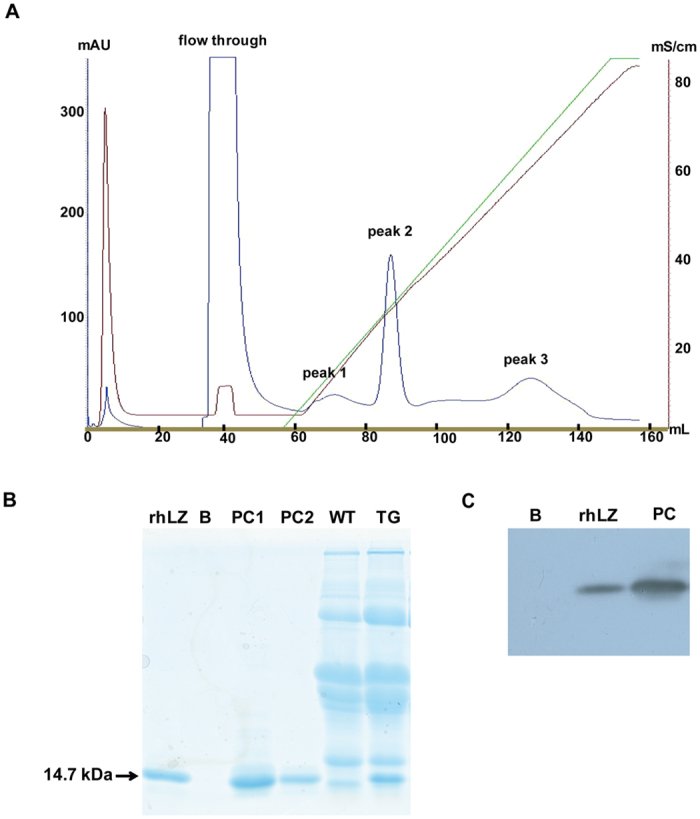
Identification of purified rhLZ from transgenic milk. (**A**) Purification of rhLZ by cation-exchange chromatogaraphy with Hiload 16/10 SP Sepharose HP column. 15 mL milk from transgenic cloned cows was loaded into a Hiload 16/10 SP Sepharose HP column. Peak 1–3, protein eluted from the colum and only the protein from peak 2 was collected. (**B**) Identification of purified rhLZ by SDS-PAGE. rhLZ, purified rhLZ from transgenic milk; B, water; PC1, 3 μg commercial rhLZ as positive control; PC2, 1 μg commercial rhLZ; WT, milk from wild type cows; TG, milk from transgenic cloned cows. (**C**) Identification of purified rhLZ by western blot. rhLZ, purified rhLZ from transgenic milk (dilution 1:10, 1 μL); (**B**) water; PC, 300 ng commercial rhLZ as positive control.

The milk samples were purified using a HiScreen SP Sepharose FF column after removal of fat and casein. As shown in Fig. 4, elution with a linear gradient of 0–1 M NaCl in 20 mM PB (pH 8.2) resulted in three peaks. The highest peak (peak 2) that contained the majority of eluted proteins was eluted at 0.4 M NaCl and collected (Fig. 4A). To further purify rhLZ, the collected fraction was concentrated using a Ultracel-30 membrane. SDS-PAGE and western blot analysis confirmed that the purified protein was rhLZ (Fig. 4B,C).

### Characterization of purified rhLZ

The molecular mass of the purified rHLZ was 14672.45 Da, as determined by MALDI-TOF-MS (Table 4). However, as compared with the molecular mass of WT human lysozyme, there was a slight difference of 1.49 Da. N- and C-terminal amino acid sequence analyses determined that the purified rhLZ was identical to hLZ from human milk (GenBank accession no. CAA32175.1). The lytic activity, as analyzed by the turbidimetric assay, was 116000 ± 8839 U/mg, which is higher than that of the commercial natural hLZ standard of 98000 ± 7352 U/mg.

### Milk yields and milk composition of transgenic cloned cow 0906

The transgenic cow 0906 gave birth and lactated naturally, and its milk yield was recorded every day. Here we showed the preliminary results from day 1 to day 17. The milk yield increased from day 1 of lactation. The concentrations of rhLZ from day 5 to day 17 were also detected and ranged from 5.19 to 8.80 g/L. The data are presented in Supplementary Table S1.

The composition of the milk from days 10–15 of lactation was tested, and the average values are presented in Supplementary Table S2. The values for fat, protein, lactose and dry matter were in the normal range of non-transgenic milk from our farm.

## Discussion

Human lysozyme is abundant in breast milk and plays a major role in the innate immune system of infants. Considering its multiple functions, rhLZ transgenic livestock as mammary gland bioreactors for large scale production of rhLZ have been developed. The first line of rhLZ transgenic goats were generated by pronuclear microinjection with a cDNA construct of hLZ driven by a 23 kb bovine as1-casein regulatory sequence. The expression level of rhLZ is 270 mg/L, which is approximately 68% of the level found in human milk^19^. Milk from transgenic and non-transgenic goats had the same fat and protein composition^19^, and the presence or expression of the transgene did not substantially impact reproductive and growth traits^26^. Recently, to meet commercial demand, two types of transgenic goats carrying the hybrid genes β-casein/hLZ and β-lactoglobulin/hLZ were generated and expressed average rhLZ levels of 2.6 g/L and 3.6 g/L in their milk, respectively^27^. Although rhLZ has been successfully expressed in goat mammary glands at high level, no previous report has described rhLZ concentrations in excess of 1.0 g/L in the milk of transgenic cows. Moreover, all previously generated rhLZ transgenic animals contained a selection marker.

Our aim was to produce transgenic cloned cows as animal mammary bioreactors with high-level of rhLZ expression in their milk. There is no doubt that an optimized construct is essential to achieve high-level expression of recombinant proteins. Our previousstudy showed that goat β-casein, as the regulatory sequence coupled to the hLZ genomic coding sequence, could produce rhLZ up to 1.4 g/L. However, the same construction failed to express rhLZ at the desired level in transgenic pigs and cows, with values of only 26 mg/L^6^ and 0.32 mg/L^28^, respectively. To improve the rhLZ expression level in milk, the expression vector pBAC-hLF-hLZ-Neo was constructed and successfully used to generate transgenic mice and pigs as models for dairy cows. Both transgenic mice and pigs produced milk with high rhLZ concentrations: 1.76 g/L of rhLZ was present in transgenic milk from mice^22^ and 2.7 g/L of rhLZ was expressed in transgenic pigs^23^. The previous results indicated that the hLF BAC regulatory region can regulate expression of exogenous genes in mice, pigs, and cows^5^,^22^,^23^. Consistent with these studies, the results of the present study confirmed that the hLF BAC regulatory region can efficiently regulate rhLZ expression in cows. The highly efficient expression of rhLZ may be attributed to two main factors. One is that hLF BAC contains all the regulatory factors necessary for high-level expression of rhLZ. The other is that the larger BAC construct had overcome the positional effect and avoided silencing of exogenous genes. Our study results also indicated that hLF BAC is an efficient construct to induce expression of milk protein in the mammary gland.

A previous study reported that disruption of the expression of neighboring genes may be induced by the existence of an antibiotic-resistance gene^29^. However, it remains unclear whether the existence of antibiotic-resistance genes is detrimental to the heath of animals and humans who consume the products from transgenic animals. In consideration of biological safety, the antibiotic-resistance gene should be removed from transgenic animals. Several methods have been developed to remove the antibiotic-resistance gene from cells used as donor cells for SCNT; the most popular of which is the Cre/loxP system. After transfer of plasmids expressing Cre recombinase and enhanced green fluorescent protein (EGFP) into fetal fibroblast from transgenic cloned cows, loxP flanked neo^R^ can be excised and the neo^R^-free fibroblast cells collected by fluorescence-activated cell sorting^30^. Considering the drawback of introducing foreign DNA vectors, which has a great potential to integrate into the genome of cells, a cell-permeable TAT-Cre recombinase was developed and the marker gene was successfully removed in goat donor cells without affecting the developmental competency of the reconstructed embryos^31^. Yu *et al.* developed a site-specific recombinase-based method. They co-electroporated phiC31 integrase mRNA and an *attB*-containing human β-defensin-3 expression vector into cells, in which excision of the selectable marker was monitored with a fluorescent double reporter^32^. However, each of these methods either require two steps to pick up positive colonies or leave another selection gene (GFP or EGFP) in the genome. In our study, we constructed a marker-free expression vector and generated transgenic colonies in a one-step process, which was both time-saving and efficient. With the development of genome editing tools such as ZFNs, TALENs and the CRISPR/Cas system, the production of transgenic animals without an antibiotic cassette offers new and better opportunities through the insertion of a gene into a specific site. Our future work will focus on the production of site-specific rhLZ transgenic farm animals.

To produce rhLZ for clinical and food applications, it is essential that the physicochemical properties of rhLZ are identical to those of naturally occurring hLZ. Purified rhLZ from transgenic rice and milk has been already reported with a similar molecular mass, isoelectric point, C- and N-terminal sequences, thermal stability, and pH stability as obtained in the present study^6^,^27^,^33^. However, there was a minor difference in the molecular mass of 1.49 Da between the natural and purified rhLZ, which could be negligible because the minimum mass increase due to post-translational modification (methylation) is 14 Da^34^. These results indicate the absence of additional post-translational modifications of rhLZ in the transgenic cows produced in this study.

The milk yield and milk composition during the course of lactation is important to assess the validity of our current approach. The increasing trend of milk yield recorded from 0906 is consistent with that of non-transgenic cows on our farm. Lactation of 0906 is good but we still need time to observe the entire 305 days of lactation. The values of the milk composition (fat, protein, lactose, and dry matter) from days 10–15 of lactation were in the normal range of non-transgenic milk from our farm. These data provide baseline experimental results, and provide a reference value for future studies. Because the milk composition is greatly influenced by the season and the feed, now it is difficult to say whether the transgenic milk composition is different from non-transgenic milk. More data are necessary for a full comparison of the milk composition and the development of a definitive conclusion.

In conclusion, we successfully generated rhLZ transgenic cows that lacked the presence of any marker gene in the genome. The rhLZ concentration in milk was as high as 3.1 g/L, which is sufficient to meet the comercial demand. The physicochemical properties of rhLZ was similar to those of naturally occurring hLZ. Hence, the results of this study lay a solid foundation for the further applications of rhLZ.

## Additional Information

**How to cite this article**: Lu, D. *et al.* Large-scale production of functional human lysozyme from marker-free transgenic cloned cows. *Sci. Rep.* **6**, 22947; doi: 10.1038/srep22947 (2016).

## Supplementary Material

Supplementary Information

## Figures and Tables

**Table 1:** Cell screening of pBAC-hLF-hLZ transfection.

Cell line	Screening method	Isolated colonies	Cell colonies ananlyzed by PCR	Freezing cell colonies	Cell colonies selected for nuclear transfer
0901FFB	Single cell amplify	298	8	8	2(#21, #27)
1003FFB	Single cell amplify	334	9	9	1(#14)

**Table 2:** Summary of nuclear transfer results.

No. of cell clone	Oocytes	Re-construct embryos	Blastocysts	Blastocysts	Recipients	Pregnancy at day 60 (%)	Born cows
0901FFB - #21	2405	1114	334	32.4	67	10 (14.9)	0814,0824,0915,0927
0901FFB - #27	1540	893	372	41.7	32	7 (21.9)	0829,0906
1003FFB - #14	1809	904	327	36.2	62	8 (12.9)	0910,0918

**Table 3:** The expression levels and enzymatic activities of recombinant human lysozyme in milk from transgenic cloned cows.

No.	The expression levels of rhLZ (mg/L)	The enzymatic activities of rhLZ (U/mL)
0814	416.05 ± 10.88	37200 ± 3153
0827	393.84 ± 11.81	25680 ± 1889
0906	3149.19 ± 24.80	305400 ± 3613
0910	2999.25 ± 19.50	129400 ± 8000

**Table 4:** Character of purifued rhLZ.

Character	hLZ	rhLZ
Molecular mass	14674.19	14672.45
N-terminal sequence	KVFERCELARTLKRL	KVFERCELARTLKRL
C-terminal sequence	VRQYVQGCGV	VRQYVQGCGV
enzymatic activity (U/mg)	98000 ± 7352	116000 ± 8839
