# Supplementary material for: Large-scale production of functional human lysozyme from marker-free transgenic cloned cows
Source: Sci Rep. 2016 Mar 10;6:22947. doi: 10.1038/srep22947 (PMC4785527; doi:10.1038/srep22947)
Supplement: Supplementary Information [file srep22947-s1.pdf]

Supplementary Information

Large-scale production of functional human lysozyme from marker-free transgenic cloned cows

Dan Lu[1,2], Shen Liu[1], Fangrong Ding[1], Haiping Wang[1], Jing Li[1], Ling Li[1], Yunping Dai[1*], Ning Li[1*]

1. The State Key Laboratory for Agro-biotechnology, China Agricultural University, Beijing , China.
2. Shanghai Institute of Medical Genetics, Shanghai Children's Hospital, Shanghai, China.

Supplementary Methods

High performance liquid chromatography (HPLC) analysis

The concentrations of rhLZ in the milk of naturally lactating transgenic cloned cow 0906 were measured by high pressure liquid chromatography (HPLC) using a Xbridge Peptide BEH C18 Column (Waters, MA, USA). Briefly, 5.0 mL milk was measured and transferred into a 15 mL centrifuge tube, and adjusted to pH 4.6 with 2 mol/L hydrochloric acid solution. After 30 min at room temperature, centrifuged immediately at 4000 rpm for 10 min in a microcentrifuge and collected supernatant into a 25 ml volumetric flask. Diluted with water to volume and filtered with a 0.45 μm membrane filter. Human lysozyme standard was purchased from Sigma-Aldrich. A 2 mg/ml standard of human lysozyme was prepared from stock standard. HPLC was performed on a Waters 1525 systerm equipped with an UV detector set at 220 nm. Samples (20 μL) were injected to the column. The flow rate was 1.0 mL/min. The concentrations were calculated according to the peak areas.

Supplementary Tables

Supplementary Table S1: Milk yield and expression level of rhLZ in the milk of transgenic cloned cow 0906.

Day of lactation	Expression of rhLZ (g/L)	Milk yield (L)
1	-	1
2	-	2
3	-	6.5
4	-	8
5	5.55	11
6	5.93	12
7	5.44	13
8	6.13	13
9	6.27	14
10	7.13	15
11	5.19	16
12	6.06	16
13	6.14	16.5
14	6.36	16.5
15	6.94	16.5
16	8.80	17
17	7.26	16.5

'-' These samples have not been determined.

Supplementary Table S2: The milk composition of transgenic milk from 0906 and non-transgenic milk.

Component (%)	Fat	Protein	Lactose	Dry matter
Transgenic milk (0906; day 10-15)	3.70 ±0.44	3.76 ±0.17	5.31 ±0.13	12.93 ±0.46
Non-transgenic milk (n=3)	3.93 ±1.06	3.10 ±0.31	4.96 ±0.25	12.69 ±1.17
Parameter range of non-transgenic milk (n>20)	1.35-6.97	2.51-3.96	4.15-5.5	9.94-15.28
